# A Review of Maricultural Wastewater Treatment Using an MBR: Insights into the Mechanism of Membrane Fouling Mitigation Through a Microalgal–Bacterial Symbiotic and Microbial Ecological Network

**DOI:** 10.3390/membranes15080234

**Published:** 2025-08-01

**Authors:** Yijun You, Shuyu Zhao, Binghan Xie, Zhipeng Li, Weijia Gong, Guoyu Zhang, Qinghao Li, Xiangqian Zhao, Zhaofeng Xin, Jinkang Wu, Yuanyuan Gao, Han Xiang

**Affiliations:** 1 School of Marine Science and Technology, Harbin Institute of Technology at Weihai, Weihai 264209, China; 23s030096@stu.hit.edu.cn (Y.Y.); 23s030098@stu.hit.edu.cn (S.Z.); lizhipengcn@hit.edu.cn (Z.L.);; 2 State Key Laboratory of Urban-Rural Water Resource and Environment (SKLUWRE), Harbin Institute of Technology, 73 Huanghe Road, Nangang District, Harbin 150090, China; 3 School of Engineering, Northeast Agricultural University, Harbin 150030, China; gongweijia@neau.edu.cn

**Keywords:** MBR, microalgal–bacterial symbiosis, maricultural wastewater, membrane fouling, microbial community

## Abstract

Membrane bioreactors (MBRs) have been utilized for maricultural wastewater treatment, where high-salinity stress results in dramatic membrane fouling in the actual process. A microalgal–bacterial symbiotic system (MBSS) offers advantages for photosynthetic oxygen production, dynamically regulating the structure of extracellular polymeric substances (EPSs) and improving the salinity tolerance of bacteria and algae. This study centered on the mechanisms of membrane fouling mitigation via the microalgal–bacterial interactions in the MBSS, including improving the pollutant removal, optimizing the system parameters, and controlling the gel layer formation. Moreover, the contribution of electrochemistry to decreasing the inhibitory effects of high-salinity stress was investigated in the MBSS. Furthermore, patterns of shifts in microbial communities and the impacts have been explored using metagenomic technology. Finally, this review aims to offer new insights for membrane fouling mitigation in actual maricultural wastewater treatment.

## 1. Introduction

The maricultural industry is one of the most rapidly developing sectors among global industries [1]. At present, the aquaculture industry is the most developed in Asia, in countries including China, Japan, India, and some southeast Asian countries (Thailand, etc.), with China accounting for about a third of global aquaculture production [2]. However, this expansion at a rapid pace has given rise to a marked increase in the discharge of effluents from mariculture and has consequently resulted in various environmental safety issues [3]. Maricultural wastewater contains high levels of organic matter, nitrogen, phosphorus, suspended solids, heavy metals, and antibiotic residues. These pollutants have contaminated aquatic environments and pose potential threats to marine ecosystems and human health [4,5]. Therefore, strengthening the routine maintenance and governance in seawater aquaculture effluent treatment facilities proves imperative [6]. Recent studies indicate that the primary pollutants found in maricultural wastewater include undigested feed, animal waste, microalgal debris, and antibiotic residues. These substances contributed to water eutrophication, disrupt aquatic ecosystems, and may pose health risks to humans through bioaccumulation [7,8]. Therefore, it is essential to develop efficient and sustainable treatment technologies to address these challenges. To address the complexities of treating actual maricultural wastewater, MBRs have increasingly garnered attention as effective wastewater treatment methods. MBRs merge conventional biological treatment processes with membrane separation technology, enabling efficient pollutant removal and solid–liquid separation. This integration demonstrates significant potential for wastewater treatment applications [9]. Compared with the traditional activated sludge method, aeration in an MBR facilitates the dissolution of soluble N_2_O into exhaust gas [10]. In MBRs, the biomass is separated using membranes, which allows for independent retention of the biomass regardless of its sedimentation properties. This results in a high concentration of biomass and a low food-to-microorganism ratio. Additionally, the unique conditions found in an MBR promote the development of a specifically activated sludge population [11]. Organic matter, nitrogen, phosphorus, and suspended solids can be removed from wastewater effectively using MBR systems. The challenge of eliminating recalcitrant organic compounds such as antibiotics can also be addressed effectively using MBRs [12]. Additionally, owing to the elevated salinity of marine aquaculture wastewater, which contrasts with the freshwater environment to which conventional activated sludge systems have been acclimated, the microbial activity is suppressed further, and the capacity for antibiotic removal is consequently diminished [13]. Zhang et al. found that MBRs independently control the sludge retention time (SRT) and hydraulic retention time (HRT), maintaining a high biomass (≥10 g MLSS L^−1^), while providing a sufficient retention time for slow-growing specific functional bacterial communities (such as Flavobacteria and Firmicutes with antibiotic degradation capabilities), thereby significantly weakening the inhibitory effect of antibiotics on microorganisms [14]. Compared with the traditional activated sludge method, MBRs have a better effluent quality, a smaller footprint, stronger resistance to shock loads, and higher efficiency [15]. This review mainly includes the following contents: (1) the mechanism of membrane fouling mitigation based on bacteria and algae; (2) membrane fouling mitigation based on the contributions of electrochemical technology; and (3) the microbial ecological network analyzed from a metagenomics perspective. This review aims to offer new insights into membrane fouling mitigation and directions for MBR use for the treatment of actual maricultural wastewater.

## 2. Membrane Fouling Mitigation Through Microalgal–Bacterial Interaction

**Figure 1:**
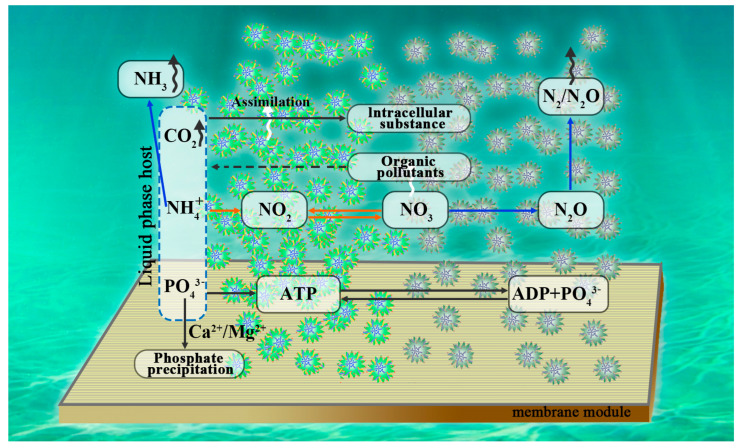
Pollutant removal in the microalgal–bacterial MBR.

Mariculture wastewater is intractable wastewater owing to its high salinity, inhibiting microbial metabolism. A biocarrier bacterial–microbial consortium and a bacterial–microbial consortium have been developed to investigate the mechanism of pollutant degradation and microbial community evolution. The biocarrier bacterial–microbial consortium exhibited excellent mariculture wastewater treatment abilities, with the highest removal for TOC (91.78%), NH_4_^+^-N (79.33%), and PO_4_^3−^-P (61.27%) [16]. Compared with traditional activated sludge, the symbiosis of bacteria and algae also demonstrates a higher pollutant removal rate and better energy-saving advantages. For instance, microalgae’s oxygen production can reduce the aeration energy consumption by 30–50%, and the particle stability can maintain a biomass retention of over 85% under hydraulic shocks [17]. The removal of the pollutants and membrane fouling mitigation between the bacteria and algae based on the MBSS are presented in Figure 1.

Microalgae promoted the degradation of polysaccharides in wastewater by enhancing microbial activity [18,19]. The increase in the protein/polysaccharide ratio tended to mitigate membrane contamination by reducing the formation of a cake layer [20]. Extracellular polymeric substances (EPSs) have been a major cause of membrane fouling in MBRs [21]. The MBSS dynamically regulated the structure and content of EPS, contributing to the reduced hydrophobicity and high aggregation of the algal sludge flocs. This further led to weak contamination adhesion on the membrane surface and mitigated membrane fouling [22]. Najm et al. explored the potential of integrating microalgae with MBR for wastewater treatment, focusing on nutrient removal, oxygen production, and carbon fixation capabilities [23]. Additionally, the symbiotic system could effectively control membrane fouling, which was a common operational issue in MBR systems. The addition of algae reduced the formation of the biofilm layer and extended the life of the membrane [24]. Furthermore, the algal–sludge bacterial-membrane bioreactor (ASB-MBR) system developed by Sun et al. [25], combining the sludge membrane bioreactor and the algae system, achieved a 25% increase in growth rate, an increase in the removal rate of COD, NH_4_^+^-N, and TN ranging from 4.6% to 10.1%, and an increase in the removal rate of PO_4_^3−^-P of 8.2%. The system has successfully reduced membrane contamination by 50%, significantly improving wastewater treatment efficiency and membrane permeability [25]. In recent years, significant progress has been made in optimizing microalgal–bacterial symbiotic membrane bioreactors system (MBSS-MBR) for nutrient removal from actual maricultural wastewater and membrane fouling control. Zhang et al. [26] found that the solids retention time (SRT) significantly impacted the characteristics of microalgae/bacteria interaction. This interaction varied with SRT due to changes in the biomass concentration and the biological community [26]. The relationship between membrane fouling and SRT was nonlinear, and the main pollutant mechanism was the formation of a gel layer [27].

Additionally, a novel membrane bioreactor (MBR) inoculated with algal–bacterial granular sludge (ABGMBR) was established by Zhang et al. [24] to improve pollutant removal and mitigated membrane fouling. There was a significant reduction in contaminants, such as particulate sludge, EPS, and soluble microbial products on the membrane. This improvement also resulted in a notable decrease in pore clogging [24]. Radmehr et al. [28] discovered that Chlamydomonas and halus have the best effects in wastewater treatment and biofuel production and are suitable for algal sludge membrane bioreactor. Compared with single algal MBR and traditional MBR, mixed algal MBR had better performance in nutrient removal, promoting chlorophyll a concentration and membrane pollution control, and bacteria dominated the membrane pollution process. The algal inoculation changed the microbial community structure and increased the lipid content [28]. The chlorophyll-a content was regarded as a key indicator of microalgal activity and biomass [29]; microalgae supplied oxygen via photosynthesis and promoted bacterial nitrification. Radmehr et al. pointed out that higher chlorophyll-a levels were associated with stronger microalgal activity, increased TN and TP removal rates, and the facilitation of larger floc formation by algal–bacterial consortia, which reduced EPS secretion and mitigated membrane fouling. Meanwhile, Rong et al. also identified the bacteria–algae ratio and found that the optimal sludge/algae inoculation ratio could optimize the treatment of actual maricultural wastewater, improve the activity of algal bacteria, and promote the formation of EPS and colloids [30]. Furthermore, Fan et al. [31] combined *Dunaliella salina* with MBR to investigate its long-term performance in treating high salinity wastewater. The results showed that MBR could effectively pretreat wastewater and the microalgae biofilm photobioreactor (MBPBR) achieved efficient nitrogen and phosphorus removal. The system operated stably, the removal rate of pollutants was high, the contribution of algae was significant, and it significantly reduced membrane fouling [31].

MBSS effectively promotes the advantages of MBR for treating maricultural wastewater, which removes nitrogen, phosphorus, total organic carbon (TOC), and antibiotics [32]. Continuous optimization of these systems, particularly through advancements in operational control and system scale-up, has facilitated broader practical applications. However, maximizing the benefits of this technology requires addressing challenges related to system complexity and salinity tolerance.

## 3. The Dual Advantages of Electrochemical Technology in the MBSS MBR

**Figure 2:**
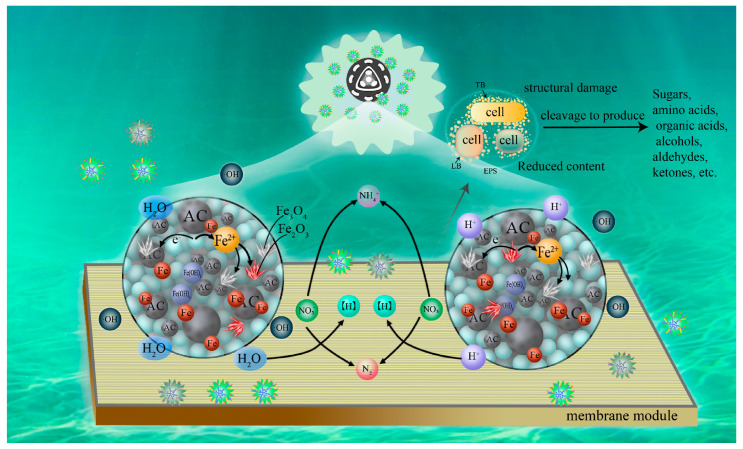
Pollutant removal in the iron–carbon micro-electrolysis MBR.

Maricultural wastewater has posed significant challenges for MBR due to its high salinity, high concentration of pollutants, and the presence of antibiotics [33,34]. The electrochemical technology (such as iron-carbon micro-electrolysis) has emerged as a promising solution (Figure 2), which tackles these issues through combining electrochemical oxidation, biological degradation processes, and membrane separation [35]. Electrochemical effects influence the organic composition and microbial community structure of SMP and play a significant role in membrane fouling and effluent quality. Analyses demonstrated that SMP was one of the primary contributors to membrane fouling in integrated systems [36]. The improvement in the scaling behavior of the MBR system was attributed to the electrophilic attack of electrochemically generated hydroxyl radicals on the electron-rich moieties of EPS organic scale [37].

The dual advantages of electrochemical-MBR (EMBR) lie in its ability to prevent biofouling by creating conditions that inhibit microbial and biofilm attachment [38]. This was accomplished through the induction of electrostatic repulsion between surfaces and foulants, modulation of ionic migration and redox potentials, and enhancement of microbial degradation of specific foulants. EMBR is defined as a novel system that couples electrochemical or bioelectrochemical units with membrane bioreactors. Its typical configuration comprises an anode chamber and a cathode chamber: on the anode side, electrogenic microorganisms oxidize organic matter in wastewater and release electrons; on the cathode side, the reduction reaction is achieved through a conductive membrane or an independent electrode, while the membrane module completes solid–liquid separation. This configuration suppresses membrane fouling, enhances pollutant removal, and simultaneously recovers energy through the use of an electric field [39]. These mechanisms reduce holistic microbial adhesion and growth, thereby mitigating membrane fouling [40], and the controlled electrochemical interaction prevents pollutants without reducing the original water treatment effect [41]. EMBR mitigates membrane fouling by generating oxidative components in situ to oxidize membrane fouling precursors [42].

Gharibian and Hazrati held that the improved pollutant behavior of the EO-MBR system was attributed to the electrophilic attack of electrochemically-generated hydroxyl radicals on the electron-rich portion of the EPS organic foulants [37]. The COD and NH_4_^+^-N removal rate reached more than 96.6% and 99.2%, respectively [43]. The trend of lower membrane fouling in EMBR systems was attributed to the in situ cleaning effect produced by oxidants on the membrane surface and the electrostatic repulsion between the membrane cathode and negatively charged contaminants [44,45]. Since irreversible pollutants were mainly caused by the penetration of small biofouling agents (especially LB/EPS), the reduced irreversible fouling could be attributed to the inhibition of the biofouling agents’ movement towards the membrane surface by electrocoagulation [46]. Additionally, applying an electric field obviously suppressed membrane fouling levels via driving off the foulants from the membrane surface or oxidizing/mineralizing pollutants deeply in the pores, mainly including the total cell, proteins, β-polysaccharides, and α-polysaccharides [47]. The cathode and anode materials in electrochemical-MBR were further investigated by Karimi et al. [48] The results showed that electrical coagulation, which was the primary mechanism contributing to EMBR, enhances membrane flux. During this process, monomers and cationic polymers of Al^3+^ and Al(OH)_2_^+^ are generated and serve as adsorbents. Negatively charged sludge and colloids are attracted to these electroactive species and precipitate, leading to a reduction in membrane fouling [48].

The electrochemical action on the membrane surface enhances biological activity in the MBR, enabling the effective removal of refractory pollutants like antibiotics [49]. The ongoing advancement of this technology, particularly in the optimization of operational parameters and the scaling-up for industrial deployment, portends promising prospective applications. Nevertheless, impediments including economic costs, electrode durability, and energy consumption necessitate resolution to facilitate broader technological adoption.

## 4. Microbial Community Analysis Based on Metagenomic Technology

### 4.1. Microbial Community Analysis in the MBR

Metagenomic research on actual maricultural wastewater supplies novel methodologies for delving into microbial community diversity, their metabolic functions, and the correlation between these elements and pollutant removal. For instance, Hong et al. employed metagenomics to reveal the co-occurrence between salt-tolerant bacterial communities and EPS gene clusters and proposed salinity-regulation strategies [50]. Xia et al. clarified, through metagenomics, the mechanism by which the MBSS system regulated the functional flora on the membrane surface to reduce membrane fouling [51]. As shown in Table 1, the microbial community in the MBR exhibits significant richness and diversity.

### 4.2. Microbial Community Diversity in Actual Maricultural Wastewater

Actual maricultural wastewater accommodates an intricate microbial community, which is pivotal for the degradation of organic pollutants, the removal of nutrients, and the dissemination of antibiotic resistance genes. Metagenomics, through direct sequencing of microbial DNA in wastewater, reveals the richness and diversity of microbial communities [61]. These microbial interactions function positively in mitigating membrane fouling [62]. Functional bacterial communities establish “albium–bacteria” or “bacteria–bacteria” symbiotic aggregates with microalgae or carrier biofilms, increasing the sludge particle size, reducing the total EPS, and alleviating membrane fouling [63]. In recent years, numerous studies employing metagenomic techniques have delineated the dynamic changes in microbial community structure within organic wastewater. For instance, Zhang et al. investigated the dynamic shifts in microbial communities during high-salinity wastewater treatment and revealed that certain bacterial phyla, such as Proteobacteria, Planctomycetes, and Bacteroidetes, were crucial for removing organic pollutants, total nitrogen, and phosphates [64]. Liu et al. pointed out that while effectively removing organic matter from the effluent, the repulsive force between pollutants and the membrane was enhanced, thereby reducing pore blockage and delaying the formation of filter cake layers [65].

### 4.3. Distribution and Transmission of Antibiotic Resistance Genes

With the increasing use of antibiotics in seawater aquaculture, ARGs in wastewater have become a focal point of concern. The dissemination of ARGs could potentially cause antibiotic resistance problems. Metagenomic technologies have offered effective methods to investigate the distribution and transmission of antibiotic-resistance genes in actual maricultural wastewater [66]. Zhou et al. found that extracellular antibiotic resistance genes (eARGs) were widely distributed in sewage treatment plants and often co-locate with mobile genetic elements, which might promote the spread of antibiotic resistance [67]. At the same time, most eARGs might originate from specific microbial hosts, such as Mycobacterium and Nitrosomonas, and there were differences between eARGs and intracellular ARGs (iARGs) hosts in urban and porcine wastewater treatment plants [68]. These findings confirmed that wastewater treatment plants were reservoirs for mobile eARGs, providing an important foundation for mitigating widespread antibiotic resistance. Copper is an essential trace element for organisms and plays a critical role in promoting animal growth and enhancing disease resistance in livestock [69]. Moreover, Cao et al. [70] realized the simultaneous and efficient removal of NO_3_^−^-N, TP, antibiotics, and Cu^2+^ from actual maricultural wastewater by using a moving bed biofilm reactor (MBBR) and discussed the toxicity of multiple antibiotics co-existing with Cu^2+^ on the growth of microorganisms and the relationship between the enzyme activity, reactive oxygen species, and microbial level on TN removal. The results showed that 70.00–94.73% of Cu^2+^ was removed by extracellular enzyme in stages I–V, and Cu^2+^ removal was mainly due to the action of extracellular enzyme. The enzymatic and non-enzymatic effects of the biofilm were also evaluated. These results provided a solid theoretical basis for the bioremediation of NO_3_^−^-N, Cu^2+^, and antibiotics in actual maricultural wastewater and promote further technological exploration and application [70].

### 4.4. Functional Analysis of Microorganisms

Maricultural wastewater contains a significant amount of organic pollutants, particularly from feed residues and metabolites generated during cultivation. The functional genes associated with organic matter degradation have been revealed, and the roles within microbial communities during wastewater treatment have been analyzed by metagenomics [71]. Lin et al. discovered that high salinity significantly affects the surface charge and EPS production in salinity-tolerant bacteria, leading to cell aggregation and enhanced biofouling development, especially under high salinity conditions, where the biofouling mechanism changes sharply from filter cake filtration to intermediate closure involving pore plugging and the cake layer formation [72]. Additionally, the pollution of microplastics (MPs) and ARGs in livestock and actual maricultural wastewater is a serious problem, but the lack of relevant research makes it challenging to formulate effective control strategies [73]. After wastewater discharge, microplastics migrate with the water flow to mangroves or adjacent bays, accumulating extensively in surface sediments due to reduced hydrodynamics, which leads to their ingestion by economic species such as oysters and prawns, resulting in bioaccumulation [74]. The traditional wastewater treatment process is not effective in removing such pollutants, and it is urgent to explore the feasibility of physical, chemical and biological methods for removal. Li et al. comprehensively discussed the current progress, challenges, and future research directions of microplastic removal and ARGs, highlighting the need for translation from the laboratory to practical applications [75].

### 4.5. Optimization of Wastewater Treatment Systems for Marine Aquaculture

Metagenomics has been utilized to analyze microbial communities within existing wastewater treatment systems and to assist in optimizing treatment processes [76]. For instance, by understanding the functional differences of microbial communities under various operational conditions, researchers have improved system designs to enhance wastewater treatment efficacy [77]. The excessive use of antibiotics has led to the spread of antibiotic resistance genes (ARGs) in aquaculture systems. Wang et al. [78] examined the profiles of ARGs in typical mariculture farms, which included both conventional and recirculating systems, using a metagenomics approach. Their investigation revealed that industrial mariculture systems serve as significant reservoirs for ARGs in coastal areas, highlighting the crucial role of recirculating systems in controlling ARG pollution [78]. Furthermore, Pratap et al. evaluated the effects of medium type (high-density polyethylene HDPE and polypropylene PPE), filling rate, and HRT on biofilm formation and moving bed–biofilm reactor (MBBR) performance in the MBBR using real municipal wastewater [79]. The results showed that the biofilm growth and the removal of organic matter and nutrients on HDPE medium were better than those on PPE medium, due to the differences in the structure and surface characteristics of the medium. Under optimal operating conditions (HRT of 6 h and fill rate of 40%), systems using HDPE media exhibit higher ammonia removal rates and more stable overall reactor performance compared to PPE carriers. A novel bacterial–algal coupling reactor (BACR) that integrates acidogenic fermentation and microalgae cultivation was first investigated by Gao et al. [80] for the treatment of mariculture wastewater. The maximum dry cell weight (DCW) of the microalgae reached 1.46 g/L. Furthermore, a co-occurrence network analysis revealed the coordination between the fermentative bacteria and microalgae in the BACR [80].

### 4.6. Discovery and Application of Novel Microorganisms

Metagenomics has significantly accelerated the discovery of previously undescribed microbial taxa, particularly within authentic maricultural wastewaters, by delivering comprehensive insights into the vast phylogenetic and functional diversity inhabiting these complex ecological niches. The functional analysis of these novel microorganisms offers the potential for designing more efficient wastewater treatment systems in the future [81]. Additionally, Park et al. [82] explored the application of the fungus-to-bacterial population inhibition strategy in MBR and found that a new fungal strain *Vanrija* sp. could effectively degrade N-acyl-homoserine lactone, a signaling molecule of Gram-negative bacteria, and after being fixed on the fluidized spheres in MBR, the biological contamination rate was significantly reduced [82]. The level of extracellular polymer material in the biofilm was reduced, and microbial communities and bacterial networks were optimized, showing great potential for the application of MBR in the treatment of acidic industrial wastewater such as semiconductor and secondary battery wastewater.

The application of metagenomics in treating actual maricultural wastewater has been crucial, which has significantly revealed microbial community diversity and the complex links to pollutant removal, offering valuable insights into maricultural wastewater treatment ecology. As metagenomic technologies advance, more in-depth findings are expected in designing and optimizing wastewater treatment systems and controlling antibiotic resistance genes.

## 5. Future Perspectives

The application prospects of MBR in the treatment of maricultural wastewater are promising, which facilitates enhancing the removal efficiency of organic matter, nitrogen, phosphorus, and antibiotics. Future research should focus on optimizing the operational parameters optimization, membrane fouling mitigation technologies, intelligent monitoring systems development, and expanding treatment targets to include emerging pollutants. Furthermore, reducing energy consumption and operational costs will encourage the widespread adoption of MBR technology for actual maricultural wastewater treatment by promoting large-scale applications and standardization.

## 6. Conclusions

This review summarized the pollutant degradation through microalgal–bacterial symbiosis for actual maricultural wastewater treatment in the MBR. The MBSS generates oxygen through photosynthesis, promoting the decomposition of organic matter by bacteria. Meanwhile, electrochemical technology triggers the generation of ·OH, enhancing the synergy between photosynthetic electron transfer in algae and the metabolic activity of bacteria and improving the high salinity tolerance of bacteria and algae. Moreover, the cake layer on the membrane surface was dynamically regulated by influencing the secretion of EPS, significantly mitigating membrane fouling and enhancing the operational stability of the system. These processes are conducive to the enrichment of dominant bacterial communities, such as the algae-promoting bacteria *Phreatobacter* sp. and *Aminobacter* sp. Meanwhile, the contents of *Verrucomicrobium* sp. and *Streptococcus* sp. were reduced, which caused membrane fouling. Microbial communities have been analyzed through metagenomics, and the composition of functional flora in the system has been regulated. This review underscores the transformative potential of MBSS in enhancing pollutant degradation and membrane fouling mitigation in MBR. This study offers new insights and perspectives of actual maricultural wastewater based on the microalgal–bacterial symbiotic MBR.

## Figures and Tables

**Table 1:** Microbial community diversity in MBR technology.

Microbial Community	Functions and Roles	Major Wastewater Types
Heterotrophic bacteria (e.g., *Pseudomonas* spp.)	Decompose organic matter, reducing BOD and COD [52]	Domestic wastewater, food processing wastewater, aquaculture wastewater
Nitrifying bacteria (e.g., *Nitrosomonas* spp., *Nitrobacter* spp.)	Convert ammonia into nitrite and nitrate, facilitating nitrification [53]	Domestic wastewater, aquaculture wastewater, nitrogen-rich industrial wastewater
Denitrifying bacteria (e.g., *Paracoccus* spp., *Pseudomonas* spp.)	Reduce nitrate and nitrite to nitrogen gas under anoxic conditions [54]	Domestic wastewater, industrial wastewater, aquaculture wastewater
Polyphosphate-accumulating organisms (PAOs, e.g., *Candidatus Accumulibacter* spp.)	Uptake and store phosphorus, removing total phosphorus [55]	Phosphorus-containing industrial wastewater, domestic wastewater
Phosphate-releasing bacteria (e.g., *certain Pseudomonas* spp.)	Release phosphate for denitrifying and PAOs’ utilization [56]	Domestic wastewater, aquaculture wastewater
Sulfate-reducing bacteria (SRB, e.g., *Desulfovibrio* spp.)	Reduce sulfate to sulfide, aiding in heavy metal precipitation [57]	Industrial wastewater (e.g., mining wastewater, petrochemical wastewater)
Methanogens (e.g., *Methanosarcina* spp.)	Convert organic matter into methane and carbon dioxide [58]	High-strength organic wastewater (e.g., slaughterhouse, food processing wastewater)
Algae (e.g., *Chlorella*, *Spirulina*)	Absorb nitrogen and phosphorus, purify water, and provide oxygen [59]	Aquaculture wastewater, domestic wastewater
Fungi (e.g., *yeasts*, *molds*)	Degrade complex organic matter and recalcitrant pollutants [60]	Industrial wastewater (e.g., pharmaceutical, textile wastewater)

Notes: Domestic wastewater originates from residential and household activities. Industrial wastewater includes effluents from pharmaceutical, food processing, petrochemical, and textile industries. Aquaculture wastewater is produced from aquaculture or livestock operations, characterized by high nitrogen and phosphorus levels. The distribution of microbial communities depends on the wastewater characteristics and operational conditions (e.g., oxygen levels, temperature, pH).
